# Preparation of Consolidated Dust Suppression Materials Based on Pectin: Graft Modification Experiment and Reaction Mechanism

**DOI:** 10.1021/acsomega.4c05299

**Published:** 2024-10-15

**Authors:** Shulei Shi, Xiao-Han Wang, Bingyou Jiang, Wenhan Tao, Chang-Fei Yu, Ben Ji

**Affiliations:** † Joint National-Local Engineering Research Centre for Safe and Precise Coal Mining, Anhui University of Science and Technology, Huainan 232001, China; ‡ Mining Enterprise Safety Management of Humanities and Social Science Key Research Base in Anhui Province, Anhui University of Science & Technology, Huainan 232001, China; § School of Economics and Management, Anhui University of Science and Technology, Huainan 232001, China; ∥ Key Laboratory of Industrial Dust Prevention and Control & Occupational Health and Safety, Ministry of Education, Anhui University of Science and Technology, Huainan 232001, China; ⊥ School of Safety Science and Engineering, Anhui University of Science and Technology, Huainan 232001, China

## Abstract

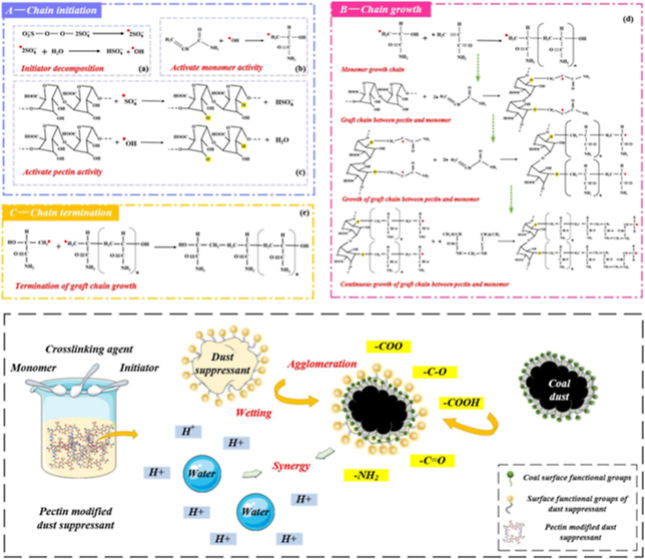

The natural material pectin was used as the matrix to prepare a dust suppressant. The regression model in response to the grafting ratio was established, and the optimum modification scheme was determined. The amount of monomer, initiator, cross-linking agent, and reaction temperature was 3.20 g, 0.20 g, and 0.15 g and 92 °C, respectively. Through Fourier transform infrared spectroscopy and X-ray photoelectron spectroscopy tests, not only the formation of the product in graft copolymerization reaction was validated but also the wettability of pectin was significantly improved. The surface morphology of pectin before and after modification was observed by a scanning electron microscope. After graft copolymerization treatment, the surface of pectin presented a dense grid structure, which proved that the pectin-modified dust suppressant can play a crucial role in wetting and condensing coal dust. Contact angle tests were used to characterize the effect of pectin modification on the wettability of bituminous coal before and after modification. The results of contact angle tests showed that when the droplets just contacted the bituminous coal flakes, the contact angle of modified pectin droplets on the flakes was the smallest, and the value was 55.21°. Compared with pure water droplets and unmodified pectin droplets, it decreased by 21.66° and 18.50°. The modification reaction process and dust suppression mechanism were explained at the molecular level.

## 1 Introduction

Pectin (PC), as a kind of polysaccharide, exists in the peels of citrus, lemon, and apple and is widely used as a natural additive in food, medicine, and health products.^1,2^ After investigation, it is found that PC resources are abundant enough. If they can be well utilized, they will produce great economic and environmental effects.^3−8^ In this research, PC was used to produce an environmentally friendly dust suppressant.

Nowadays, people pay more and more attention to health and ecological issues.^9−14^ High concentration of dust in coal mine production not only endangers the safety and health of workers but also causes irreversible dust pollution.^15−19^ The studies presented here show that chemical dust suppressants are effective methods for dust pollution. Scholars have carried out many studies on the preparation and optimization of dust suppressants.^20,21^ When dust suppressants are applied in a coal mine, workers are inevitably exposed to them. Therefore, the research of low toxic and economical dust suppressants has become a hotspot.^22−25^ Bao et al. made a chemical modification of bentonite and prepared a new dust suppressant through graft copolymerization, which verified that the dust suppressant can solve the problems of coal dust pollution and water waste.^26^ Fan et al. generated a macromolecular product by cross-linking sodium lignosulfonate and acrylamide (AM).^27^ Zhou et al. prepared an effective dust suppressant by extracting cellulose from bagasse.^14^ Zhang et al. developed a degradable dust suppressant with natural polymer guar gum.^28^ Jin et al. synthesized a dust suppression product by a modification of soybean isolate protein.^29^ Liu et al. determined four optimal modification conditions through single-factor experiments and made a high-efficiency dust suppressant.^30^

Scholars have made important contributions to research on dust suppressants. However, it is worth noting and improving that the mechanism of dust suppression is not clear and the field application is restricted.^31−34^ In addition, we must face the economic effects and component safety of dust suppressants. This study used PC as the graft skeleton to prepare a new environmentally friendly dust suppressant named PC-*g*-AM/TO-10. Through experimental research and molecular reaction mechanisms, the dust suppression mechanism of the product was explained. This research can provide a reference for subsequent studies, which can promote the innovation of dust suppressants with healthy, environmental, and economic properties.

## 2 Experiments

### 2.1 Materials

The matrix used in the experiment is PC, the monomer is acrylamide (AM), the initiator is potassium persulfate (KPS), and the cross-linking agent is *N*,*N*′-methylene bisacrylamide (MBA). All of the chemical materials used in this study are of analytical grade. The reagent information is shown in Table 1. The bituminous coal samples involved in this paper come from the Dalita Coal Mine in Shaanxi Province.

**Table 1:** Reagent Information

number	name of reagent	production company	reagent abbreviation
1	Pectin (LA-S10)	Schwartz, Italy	PC
2	Acrylamide	Aladdin Co.	AM
3	Potassium persulfate	Aladdin Co.	KPS
4	*N*,*N*′-methylene bis(acrylamide)	Aladdin Co.	MBA

### 2.2 Modification Process

**Figure 1:**
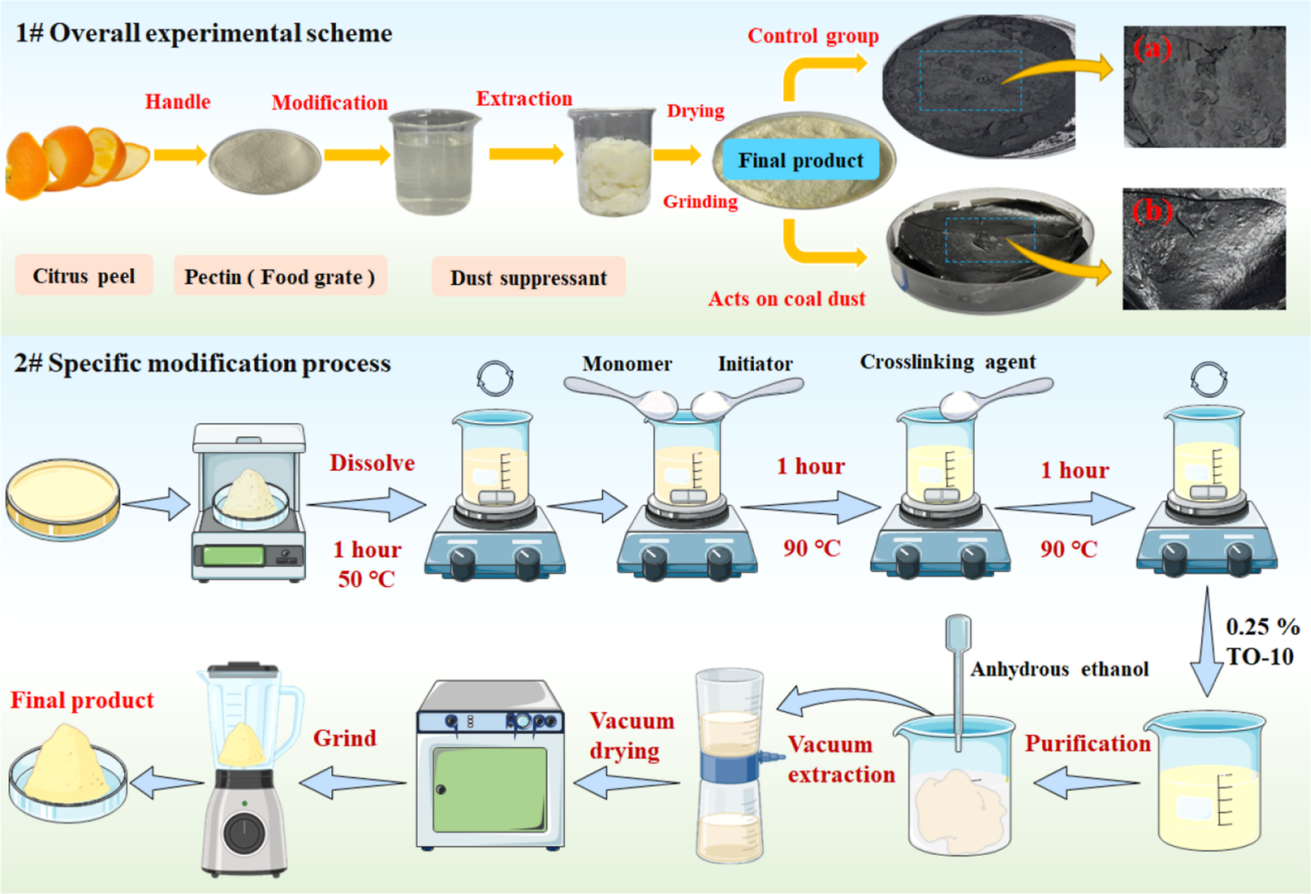
Process diagram of modification experiment [1# overall experimental scheme, (a): control group of dust suppression experiment, (b): coal acted by dust suppressant, 2#: specific modification process].

Figure 1 shows the process diagram of modification. 1 g of PC was completely dissolved in 150 mL of pure water. After adding KPS and AM to the solution, the mixture was stirred in a water bath at 90 °C for 1 hour, then MBA was added and stirred for 1 hour to obtain the cross-linked product PC-*g*-AM. After the TO-10 solution (0.25 wt %) was added to the mixture, the final product was named as PC-*g*-AM/TO-10.

**Figure 2:**
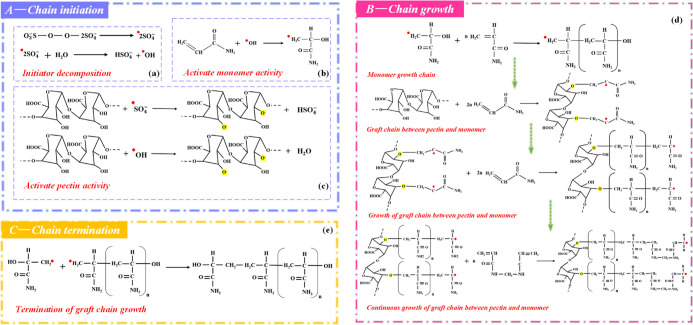
Modification reaction process (a) chain initiation, (b) chain growth, (c) chain termination.

The modification reaction process is shown in Figure 2. PC contains a large number of reactive side groups (such as hydroxyl, ester, and carboxyl). The modification of PC can be divided into four stages: activation, chain initiation, chain growth, and chain termination.^29^ Under the conditions of 50 °C and initiator, the −OH groups in PC are broken and the graft active sites are effectively stimulated. In the chain initiation stage, the initiators generate SO_4_^–^ and form free hydroxyl radicals under the heating conditions. The initiators not only promote the breakage of C=C but also excite free radicals on the PC molecule available for reaction. In the chain growth phase, on the one hand, AM forms a graft chain for growth. On the other hand, the polymerized AM branched chains are grafted to the active sites of the main chain. Under the condition of the initiator, the double bonds of the MBA molecules are broken and grafted onto the chains of AM, which act as grafting bridges to connect and expand the active chains. Chain termination is the final stage of grafting. When the chain growth points disappear, the kinetic chains are terminated and the graft copolymer (PC-*g*-AM/TO-10) is formed.

### 2.3 Response Surface and Optimization Design

The whole modification process was divided into three stages: preliminary screening, response optimization, and final verification. Based on pre-experiments, AM, KPS, MBA, and reaction temperature were classified as the main factors in the single-factor experiment. The amount of each component and the reaction conditions must be further optimized. In this work, the Box–Behnken design (BBD) in the RSM was used to carry out further response analysis. Based on the single-factor experimental results, four factors and three levels of BBD were chosen, as shown in Table 2.

**Table 2:** Design of Test Factors and Levels

level	AM (g)	KPS (g)	MBA (g)	temperature (°C)
–1	2	0.15	0.10	80
0	3	0.20	0.15	90
1	4	0.25	0.20	100

To quantify the modification effect, the experimental products were treated with an ethanol absolute solution and a vacuum filtration device. The final products were dried until the mass no longer changed. According to the mass relationship between the final product and PC, the corresponding grafting ratio was calculated.^29,30^ The grafting ratio *G* % under different modification conditions is defined by eq 1
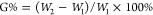
1where *W*_1_ represents the mass of PC. *W*_2_ represents the mass of the final product PC-*g*-AM/TO-10, and the unit is g. Through multifactor response analysis, the optimal dosages of the components and the experimental conditions were determined. Meanwhile, the response analysis was validated by further experiments. The above calculations and analysis were performed by Design-Expert software.^35,36^

### 2.4 Response Surface Model

The multivariate response regression model is established by the quadratic polynomial shown in eq 2
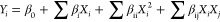
2where *Y*_*i*_ represents the response value (Grafting ratio). *X*_*i*_ and *X*_*j*_ denote the independent variables (*i* ≠ *j* ϵ [1,4]). β_0_ is the constant term coefficient. β_*i*_ is the linear effect coefficient. β_*ii*_ is the quadratic effect coefficient. β_*ij*_ is the interaction effect coefficient.^37^

### 2.5 Characterization Tests

FTIR spectra were recorded with a solid–liquid two-phase infrared spectrometer (Bruker Scientific Technology Co.). XPS spectra were measured by X-ray photoelectron spectroscopy (Thermo Fisher Scientific Co.). Combining FTIR and XPS spectra, the contents of different elements and corresponding functional groups can be analyzed. The SEM (Hitachi Co.) was used to observe the surface of the products under different magnification conditions (200, 500, and 2000 times). At the same time, dust suppression experiments were carried out; the coal sample was soaked in a modified dust suppressant, and another group of pure water was set as a control.

### 2.6 Experimental Characterization of Dust Suppression

0.20 g amount of bituminous coal was prepared into coal flakes at a pressure of 20 MPa, and the dust suppression solutions before and after PC modification were prepared for follow-up experiments. A contact angle measuring instrument (DSA30) was used to measure the changes of contact angles of dropping water solution, PC solution, and modified PC solution on the surface of bituminous coal flakes. Each set of contact angle experiments was repeated three times, and the average values were taken as the final contact angle values.

## 3 Results and Discussion

### 3.1 Analysis of Single-Factor Experiment

**Figure 3:**
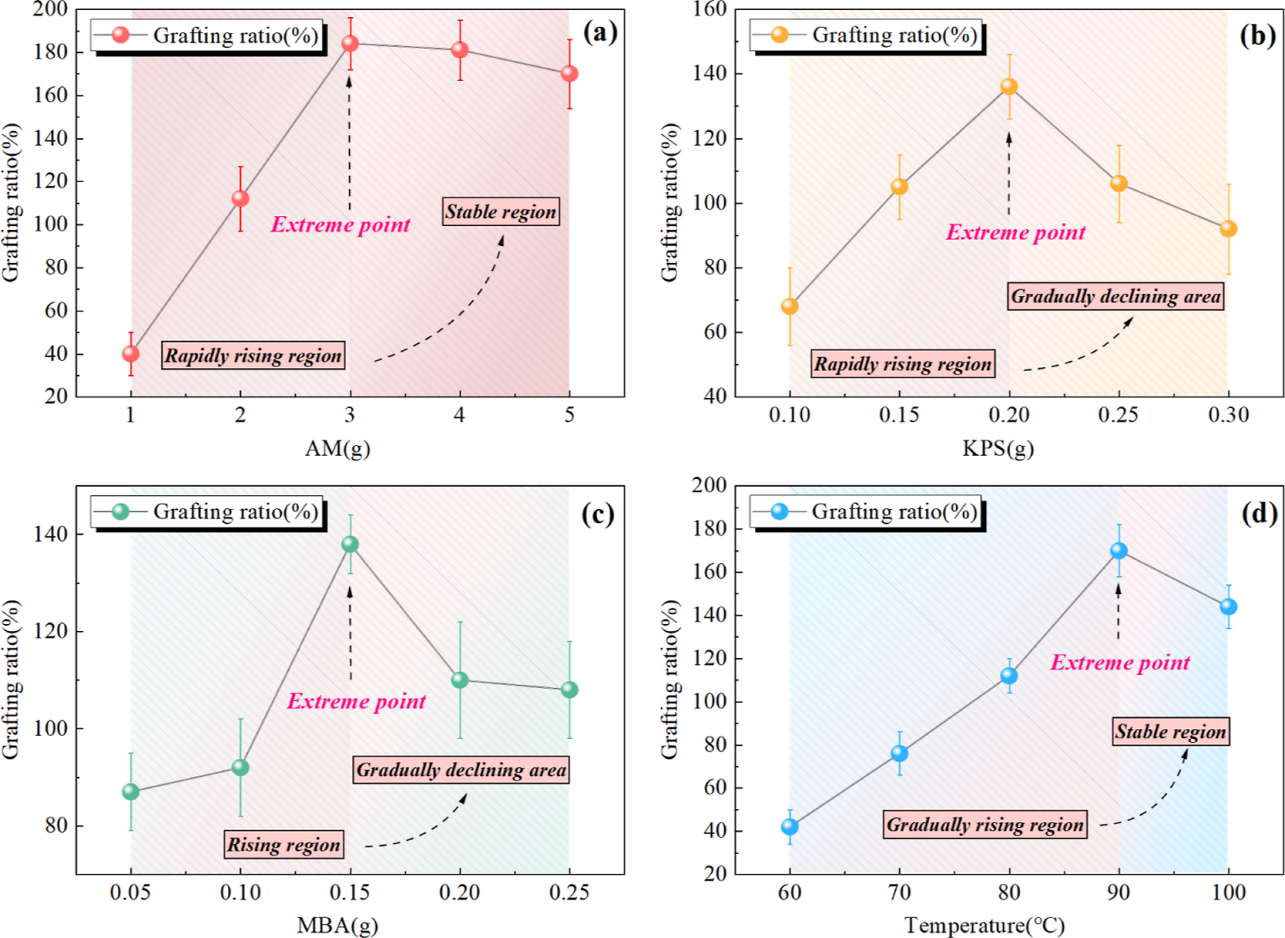
Effect of single-factors on grafting ratio (a) AM, (b) KPS, (c) MBA, (d) temperature.

The influence of single-factors on the modification process was studied by the grafting ratio. As shown in Figure 3a, with the increase in AM dosage, the grafting rate tends to increase first and then decrease. When the amount of AM is 3 g, the grafting ratio is the maximum. It can be seen that the content of AM is an important factor that affects the modification process. The insufficient amount of AM results in less exposure to free radicals, which leads to poor grafting effects. With the increasing amount of AM, the probability of monomers colliding and combining with each other increases, which weakens the growth ability of the main chain.

As shown in Figure 3b, when the mass of KPS reaches 0.2 g, the maximum grafting ratio reaches 136%. At the initial stage, insufficient KPS leads to a limited number of active sites. As the mass concentration of KPS increases, there are sufficient free active sites on the macromolecules, which is favorable for the growth of the grafted chain. However, when the amount of KPS reaches a certain value, the excessive free radicals are unfavorable for the graft copolymerization reaction and the growth of the main chain.^14^ More seriously, it will lead to the early termination of the graft copolymerization reaction.

Figure 3c displays the relationship between the MBA content and the grafting effect. The function of the MBA is to link the polymer. When the amount of MBA is insufficient, the structure of the graft copolymer is unable to achieve the saturation state. When the amount of MBA is excessive, it tends to result in the competition of grafting sites. According to the above experiments, when the dosage of MBA is 0.15 g, the modification effect is optimum.

Figure 3d shows the effect of the temperature on graft copolymerization. The grafting rates grow with the increase of the temperature when the temperature is lower than 90 °C. High temperature increases the activity of molecules, which tend to participate in the graft copolymerization reaction and effectively enlarge the main chain length. However, a high temperature can promote the homopolymerization of monomers and eventually lead to the termination of graft copolymerization.^30^

In this job, the reasonable ranges of values are preliminarily selected through single-factor experiments.^38^ The AM is 2–4 g, the KPS is 0.15–0.25 g, the MBA is 0.10–0.20 g, and the reaction temperature is set at 80–100 °C. The further experimental design (BBD) was established based on the results of single-factor experiments.

### 3.2 Response Surface and Residual Normal Probability Analysis

The BBD was applied to determine 29 sets of experimental protocols, and the experimental designs and results are shown in Table 3.

**Table 3:** Design and Result of BBD Experiment

		factors			response	
std	run	AAM (g)	KPS (g)	MBA (g)	temperature (°C)	grafting ratio (%)
19	1	2	0.20	0.20	90	79
9	2	2	0.20	0.15	80	89
20	3	4	0.20	0.20	90	120
12	4	4	0.20	0.15	100	123
1	5	2	0.15	0.15	90	91
6	6	3	0.20	0.20	80	102
3	7	2	0.25	0.15	90	88
23	8	3	0.15	0.15	100	126
25	9	3	0.20	0.15	90	160
21	10	3	0.15	0.15	80	124
4	11	4	0.25	0.15	90	112
18	12	4	0.2	0.10	90	120
24	13	3	0.25	0.15	100	144
22	14	3	0.25	0.15	80	116
29	15	3	0.20	0.15	90	170
28	16	3	0.20	0.15	90	168
26	17	3	0.20	0.15	90	172
15	18	3	0.15	0.20	90	106
2	19	4	0.15	0.15	90	112
13	20	3	0.15	0.10	90	102
8	21	3	0.20	0.20	100	128
5	22	3	0.20	0.10	80	104
27	23	3	0.20	0.15	90	176
10	24	4	0.20	0.15	80	109
11	25	2	0.20	0.15	100	93
17	26	2	0.20	0.10	90	92
14	27	3	0.25	0.10	90	100
16	28	3	0.25	0.20	90	104
7	29	3	0.20	0.10	100	120

**Figure 4:**
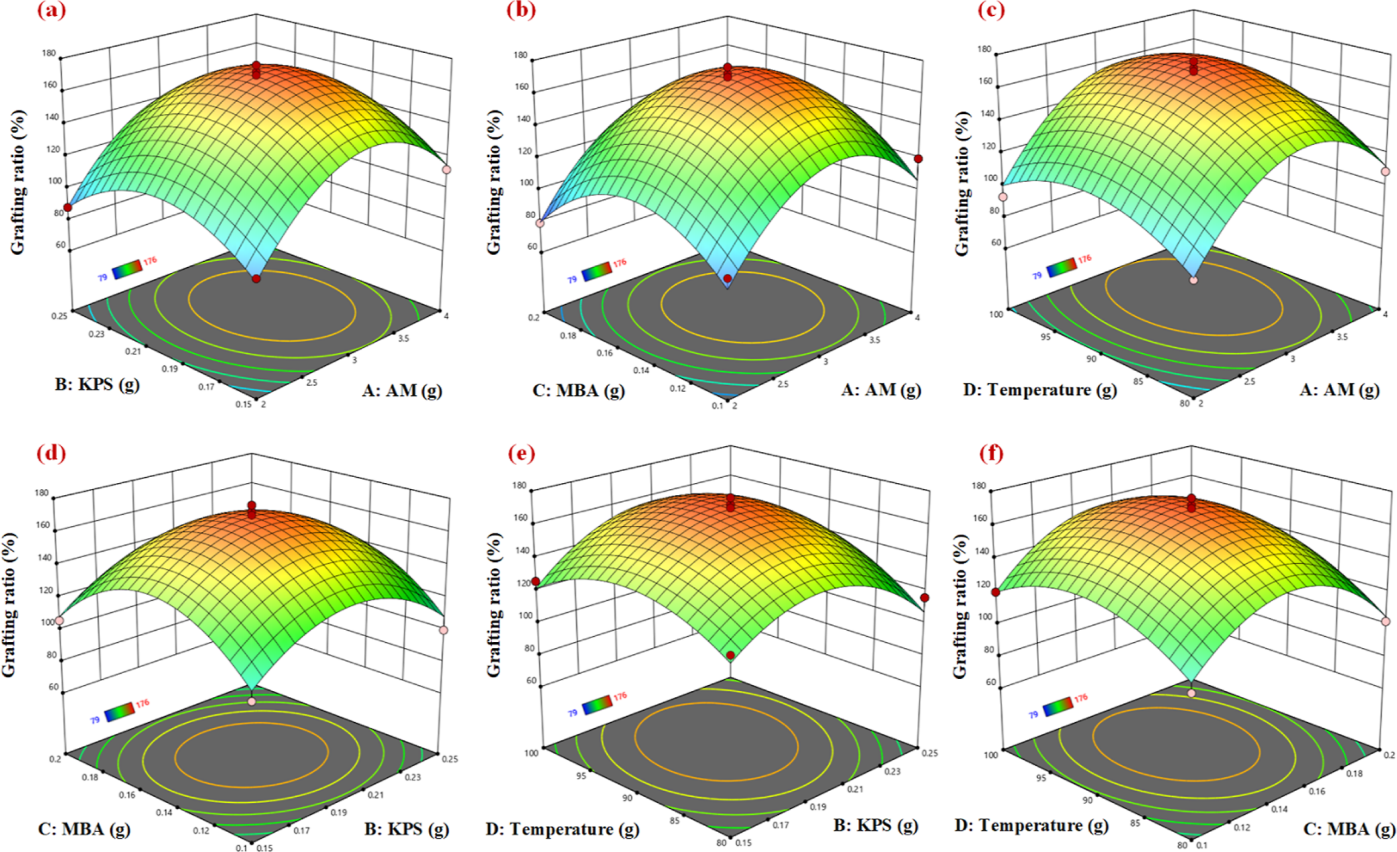
Quadratic response surface diagram (a) AM and KPS factors, (b) AM and MBA factors, (c) AM and temperature factors, (d) KPS and MBA factors, (e) KPS and temperature factors, (f) MBA and temperature factors.

Based on the BBD experimental results, the response surface and contour figures were generated, which can visualize the effect of the interaction between the four factors on the grafting ratio, as shown in Figure 4. Meanwhile, the interaction effects of different factors can be derived from the figures.^29,37^

**Figure 5:**
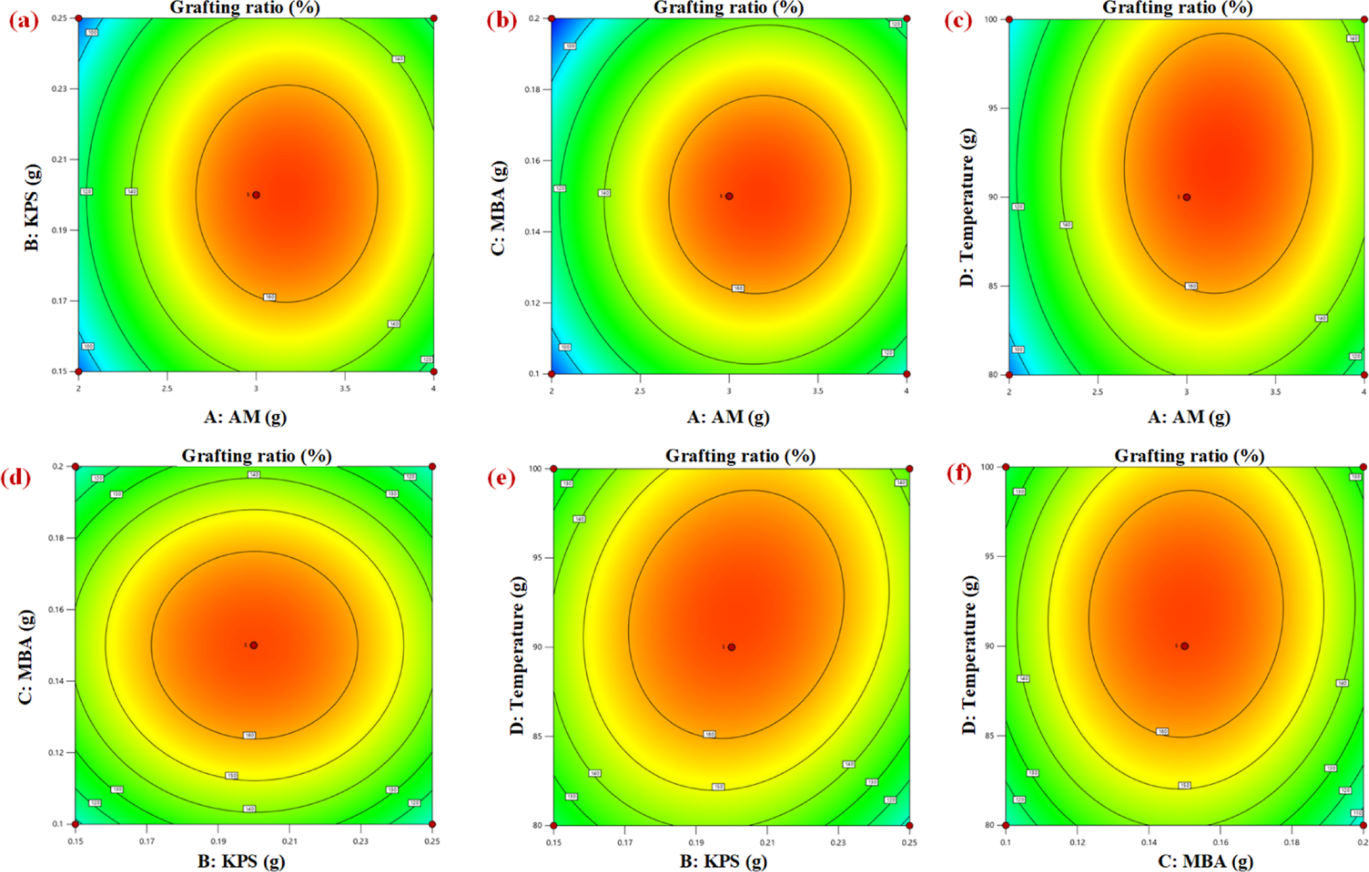
Quadratic response cloud diagram. (a) AM and KPS factors, (b) AM and MBA factors, (c) AM and temperature factors, (d) KPS and MBA factors, (e) KPS and temperature factors, (f) MBA and temperature factors.

Figures 4a and 5a show the interaction effect between AM and KPS on the response of the grafting rate. Appropriately increasing the contents of AM and KPS have positive effects on the grafting ratio, but the continued increases have negative effects. The unnecessary homopolymerization is caused by the excess of monomers, which drastically reduces the effective grafting sites. As shown in Figures 4b and 5b, there is an obvious interaction between the AM and MBA. It can be demonstrated that the MBA, as the bridge of graft copolymerization, assumes the important role of growing the main chain. The appropriate amounts of MBA and AM can effectively generate the modified products.

However, the interaction between KPS and MBA is not significant, as shown in Figures 4d and 5d. By a comparison of Figures 4 and 5, the interaction between the reaction temperature and KPS is the strongest. Because KPS is a class of compounds that can be easily decomposed by heat to form free radicals and can initiate free radical polymerization reactions and cross-linking reactions between molecules. In addition, an appropriate reaction temperature can induce a large number of free radicals available for grafting, which can have positive effects on the modification reaction.

According to the regression model, AM and the reaction temperature are essential factors. Among the interaction terms of the model, the KPS and reaction temperature have a significant influence. Based on the RSM analysis, the optimal dosages and reaction conditions were predicted. The grafting ratio is 171.17% when AM is 3.18 g, KPS is 0.20 g, MBA is 0.15 g, and the temperature is 91.97 °C. To make the experiments more operable, the optimal conditions are modified to 3.20 g, 0.20 g, 0.15 g, and 92 °C, respectively. The relative error between the measured and predicted grafting rates was 0.87%. The model is feasible and can precisely predict the experimental results.

### 3.3 Quadratic Regression Model and ANOVA Analysis

RSM is used to optimize and exploit quadratic polynomial equations in response to the grafting ratio, which can accurately analyze the interactions between different variables.^39^ The regression model with grafting rate as the response was obtained, as shown in eq 3. The ANOVA results of the quadratic model are shown in Table 4
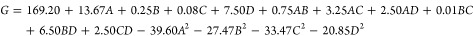
3

**Table 4:** ANOVA of Quadratic Regression Model for Grafting Efficiency Response

source	sum of squares	d*f*	mean square	*F*-value	*p*-value	
model	19977.58	14	1426.97	20.44	<0.0001	significant
A-AM	2241.33	1	2241.33	32.10	<0.0001	
B-KPS	0.7500	1	0.7500	0.0107	0.9189	
C-MBA	0.0833	1	0.0833	0.0012	0.9729	
D-temperature	675.00	1	675.00	9.67	0.0077	
AB	2.25	1	2.25	0.0322	0.8601	
AC	42.25	1	42.25	0.6052	0.4496	
AD	25.00	1	25.00	0.3581	0.5591	
BC	0.0000	1	0.0000	0.0000	1.0000	
BD	169.00	1	169.00	2.42	0.1420	
CD	25.00	1	25.00	0.3581	0.5591	
A^2^	10171.85	1	10171.85	145.70	<0.0001	
B^2^	4896.49	1	4896.49	70.14	<0.0001	
C^2^	7268.60	1	7268.60	104.12	<0.0001	
D^2^	2819.82	1	2819.82	40.39	<0.0001	
residual	977.38	14	69.81			
lack of fit	836.58	10	83.66	2.38	0.2096	not significant
pure error	140.80	4	35.20			
cor total	20954.97	28				

The reliability analysis of the regression variance is shown in Table 5. The significance and validity of the regression model can be evaluated by the values of characteristic parameters.^40,41^ Based on the ANOVA of the regression model, the *p*-value is significant (*p*-value < 0.0001), the *p*-value without fitting is not significant (*p*-value = 0.2096), and the *F*-value of the model is 20.44. These parameters prove that the response model predicted values fit well with the tested values.^42^ The *R*^2^ and adjusted *R*^2^ of the regression model are 0.9534 and 0.9067, respectively, indicating that the model has good compatibility.^43,44^ The coefficient of variation (C.V. %) of the model is 7.02% (<10%), which indicates the validity and feasibility of the model data.^36^

**Table 5:** Reliability Analysis of Variance of Quadratic Response Model

*R*^2^	adjusted *R*^2^	predicted *R*^2^	C.V. %
0.9534	0.9067	0.7595	7.0200

### 3.4 Analysis of Structural Characterization before and after Modification

**Figure 6:**
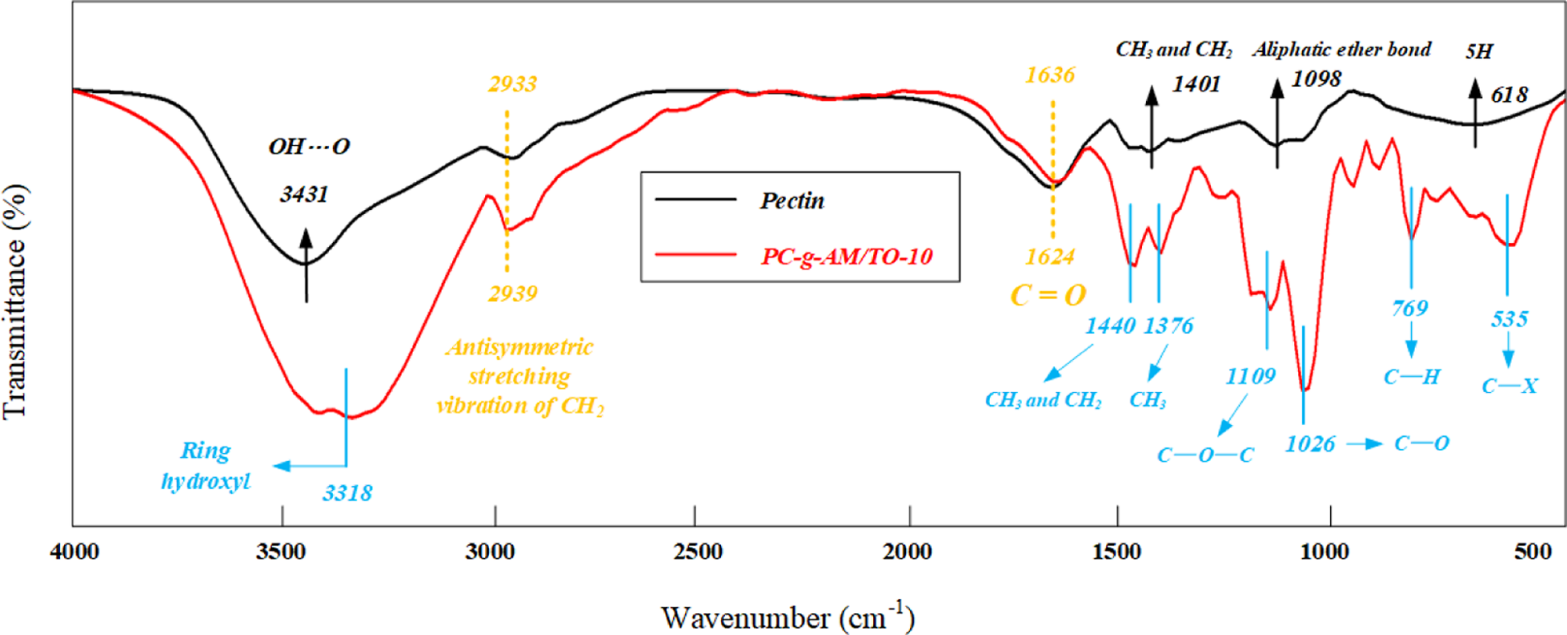
FTIR spectra of pectin and PC-*g*-AM/TO-10.

Figure 6 exhibits the FTIR spectra of PC and the modified product. In the spectrum of the product, PC changed from hydroxyl polar bond to cyclic hydroxyl before and after modification. The absorption peak at 1109 cm^–1^ was observed, accompanied by the stretching vibration of the C–O–C group. There was an obvious absorption peak at 1026 cm^–1^, accompanied by stretching vibration of the C–O group.^45^ The absorption peaks of the C–H and C–X groups appeared at 769 cm^–1^ and 535 cm^–1^, indicating that AM and MBA participated in the graft copolymerization process.^29,46^ Throughout the modification process, the C=C group and the hydroxyl group were destroyed under the action of the KPS, and NH_2_ and C–O groups were introduced into the product.^3,47−49^ In addition, a graft copolymerization reaction occurred in the system under the conditions of KPS and high temperature, which resulted in the generation of a new substance.

**Figure 7:**
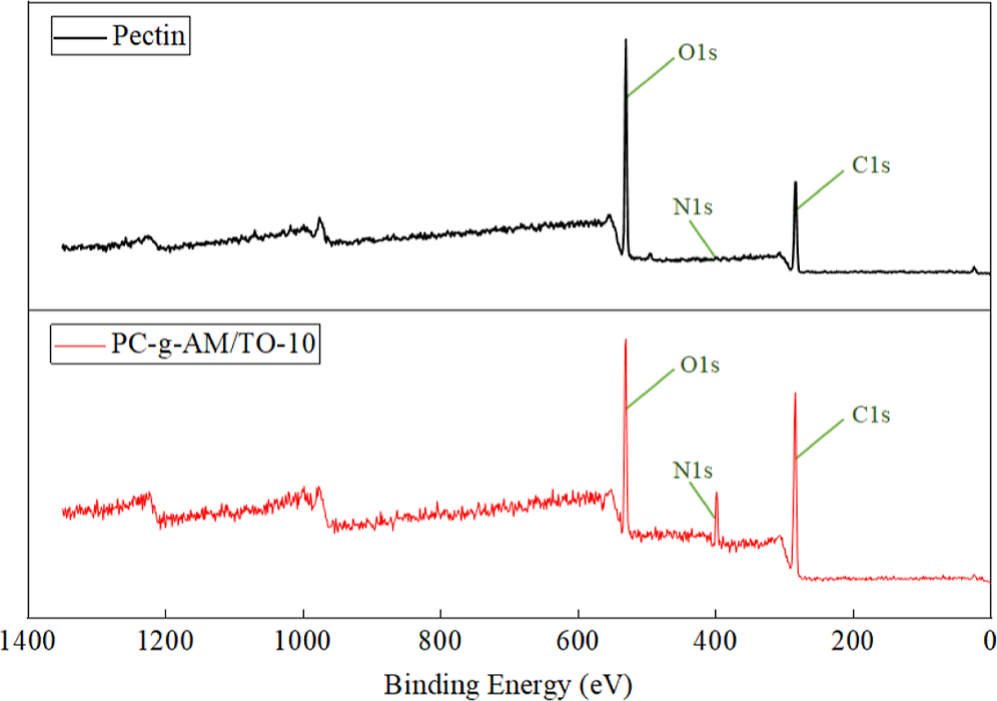
XPS spectra of pectin and PC-*g*-AM/TO-10.

The generation of modified products was determined based on the changes in FTIR spectra. To further confirm the changes in the content of different elements and functional groups in products, the experiments were carried out, and XPS spectra were obtained, as shown in Figure 7.

**Figure 8:**
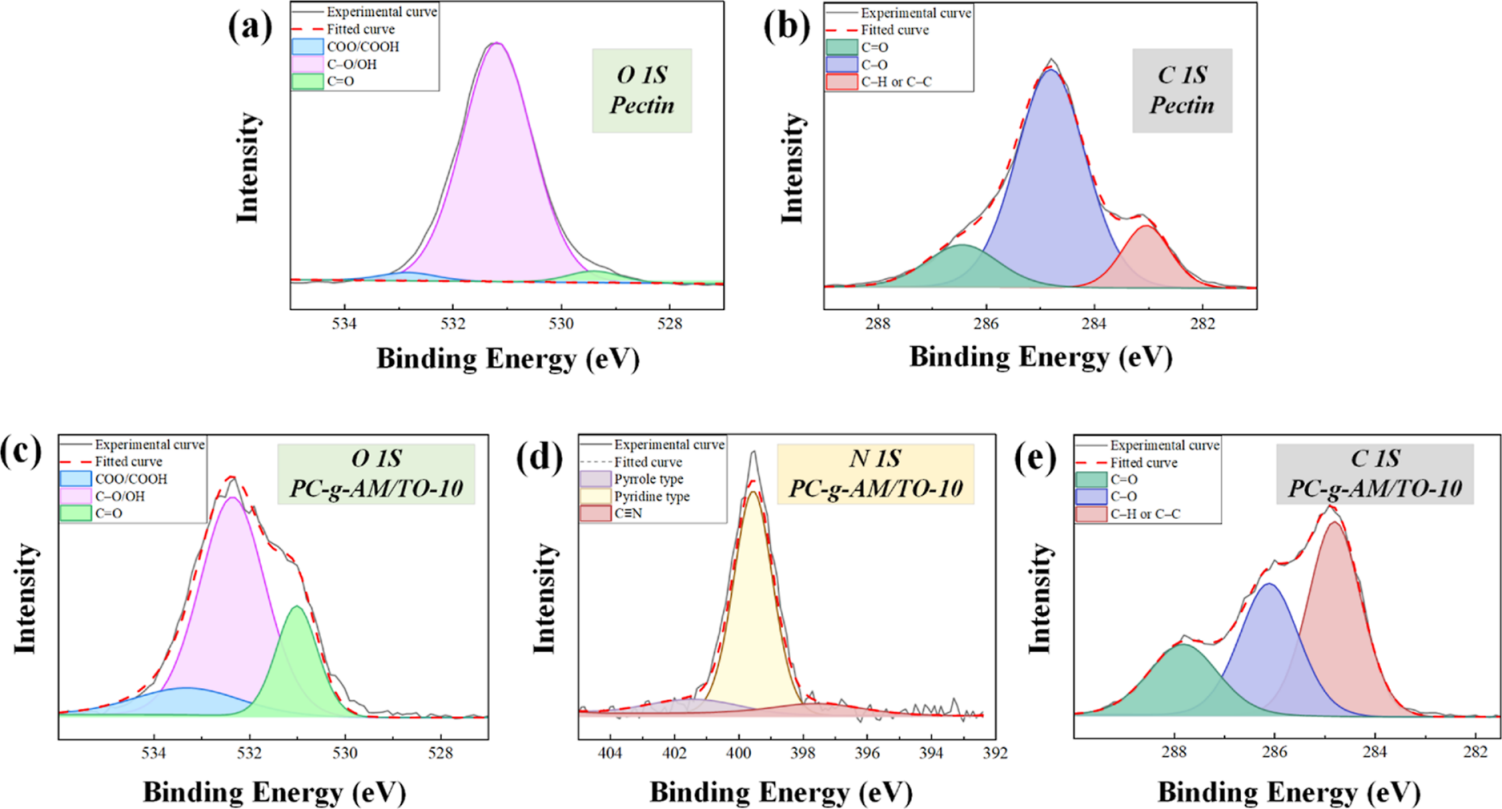
XPS fitting curves of pectin and PC-*g*-AM/TO-10 (a) O 1s pectin, (b) C 1s pectin, (c) O 1s PC-*g*-AM/TO-10, (d) N 1s PC-*g*-AM/TO-10, (e) C 1s PC-*g*-AM/TO-10.

The obvious peaks in the XPS spectra of PC are the C 1s and the O 1s peaks. In the XPS spectra of the modified PC, in addition to the corresponding peaks of C 1s and O 1s, an additional peak was found at N 1s, which indicated that related functional groups are introduced after the PC modification. The elemental contents are listed in Table 6. The variation of the characteristic peaks of functional groups was obtained by peak fitting of the XPS spectrum, as shown in Figure 8. The corresponding functional groups were determined according to the position of each characteristic peak after fitting, and the area of each characteristic peak was calculated to obtain the content ratio.^50−53^ The C 1s and the O 1s peaks were calculated, and the results are listed in Table 7.

**Table 6:** Content of Elements in XPS Spectra

	element content (%)		
test sample	C	N	O
pectin	54.49	-	45.51
PC-*g*-AM/TO-10	63.52	9.75	26.74

**Table 7:** XPS C 1s and O 1s Data of Pectin and PC-*g*-AM/TO-10

		pectin		PC-*g*-AM/TO-10	
	attribution	binding energy (eV)	content (%)	binding energy (eV)	content (%)
elemental peak	C–H/C–C	283.04	71.59	284.80	44.83
	C–O	284.80	14.09	286.11	34.00
	C=O	286.44	14.32	287.81	21.17
O 1s	C=O	529.40	3.40	531.00	21.09
	C–O/–OH	531.18	93.99	532.35	65.72
	COO/COOH	532.83	2.61	533.30	13.19

The decisive factors for assessing the wettability of the final product on coal are majorly the content of hydrophobic and hydrophilic functional groups.^54^ According to the results of the split-peak fitting of C 1s spectra, the content of C–H/C–C groups accounted for 71.59% of the original PC, and this component accounted for 44.83% of the modified PC. Compared to the original PC, the reduced group content of 26.76% was partially converted to other group contents. Interestingly, the C–O and C–O groups in the modified PC increased by 19.91% and 6.85% compared with the original PC. A large number of C–O groups and C=O groups were introduced into the products. The results of the O 1s split-peak fitting were observed, and there were three different types of oxygen atoms. Oxygen elements mainly exist as C–O and –OH in the original and modified PC, accounting for 93.99% and 65.72%, respectively. The C=O and COO/COOH groups in the modified PC increased by 17.69% and 10.58% compared with those in the original PC. A large number of C=O and COO/COOH groups were introduced into the products, resulting in a decrease in the proportion of C–O/–OH.

From the comprehensive characterization, the introduction of wettability groups (C=O, COO/COOH and NH_2_) into the PC is the essence of the modification.^54−56^ The decomposition of KPS increases the number of free radicals of PC, which triggers more graft binding sites. By the action of the MBA, the graft copolymerization products are induced to form a more extensive network structure so that the growth of the modified chain is effectively promoted.

### 3.5 Analysis of Surface Morphology before and after Modification

**Figure 9:**
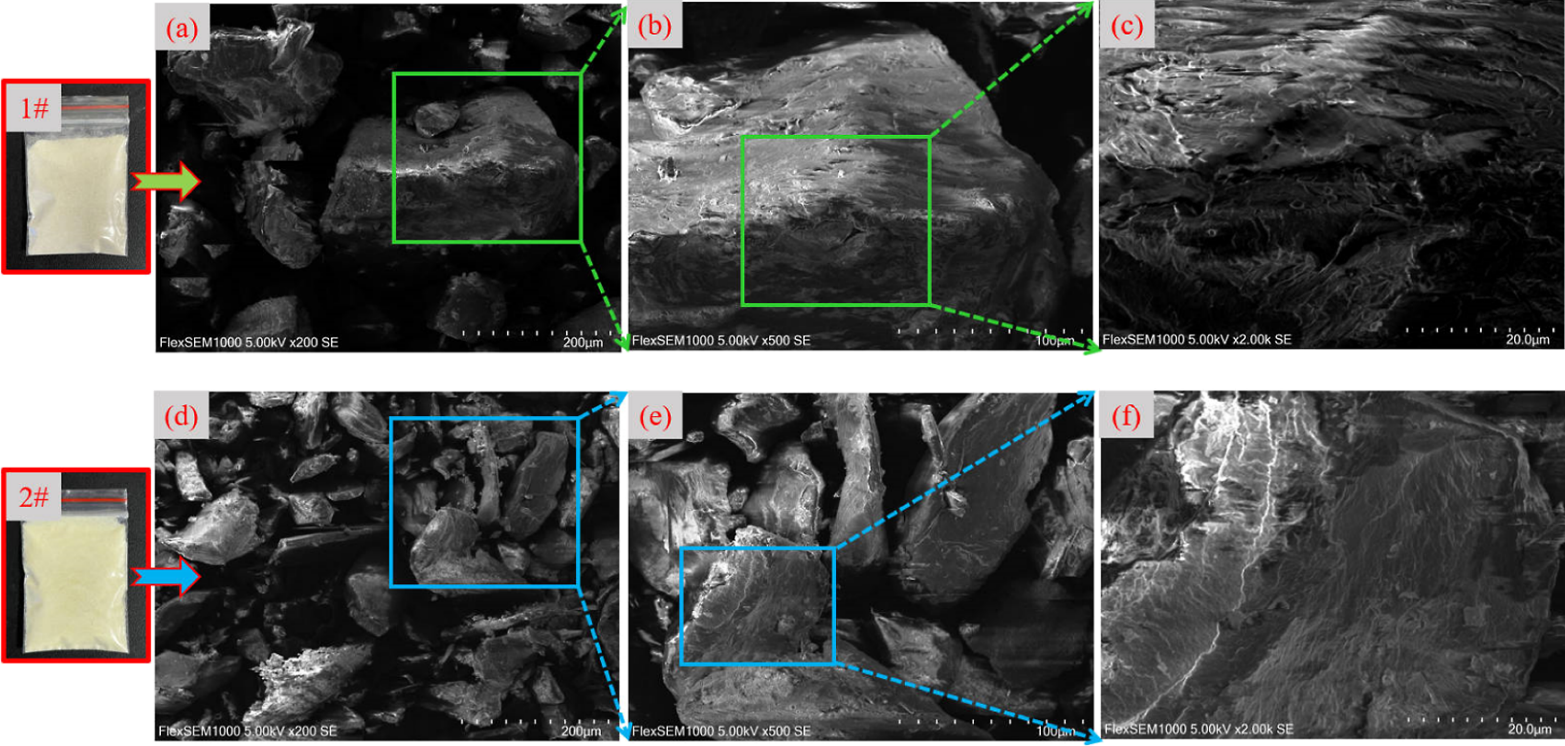
SEM scanning diagrams of pectin before and after modification [1# pectin, (a) 200 times, (b) 500 times, (c) 2000 times, 2# modified pectin, (d) 200 times, (e) 500 times, and (f) 2000 times].

To better visualize the changes in the microstructure of the PC surface before and after modification, Figure 9 displays the SEM images of the PC under different magnification conditions.

There is a significant change in the color of the particles before and after modification (1#–2#). As shown in Figure 9a,c, the surface of an unmodified PC is relatively smooth, with few pores and cracks. In addition, the distance between particles on the PC surface is larger, which has a weaker ability to capture coal dust particles.^57^

As shown in Figure 9d,f, the pore structure on the surface of the modified PC is more obvious and irregular, and part of it appears to be a laminar structure. Most of the areas are composed of irregular protrusions and pores, which implies that the surface of the modified PC is rough. When the magnification is 500 times, the bedding structure of the surface is highlighted, which can be related to the formation of a grafting and cross-linking network. When the image is further enlarged to 2000 times, it can be found that the surface of the modified PC is dense and nonporous, which can effectively capture the free coal dust particles. Therefore, after modifying the PC, the surface appears more and denser porous 3D network structure.^58,59^ Large-scale pores and laminar folds can effectively increase the contact area with coal dust particles, they can encapsulate and agglomerate coal dust and achieve the effect of reducing coal dust.^47^

### 3.6 Analysis of Surface Morphology before and after Modification

**Figure 10:**
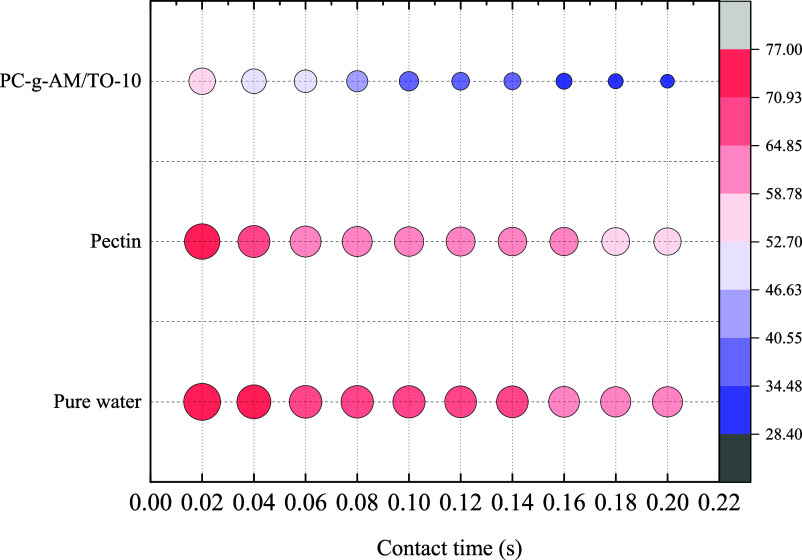
Experimental test values of contact angle.

**Figure 11:**
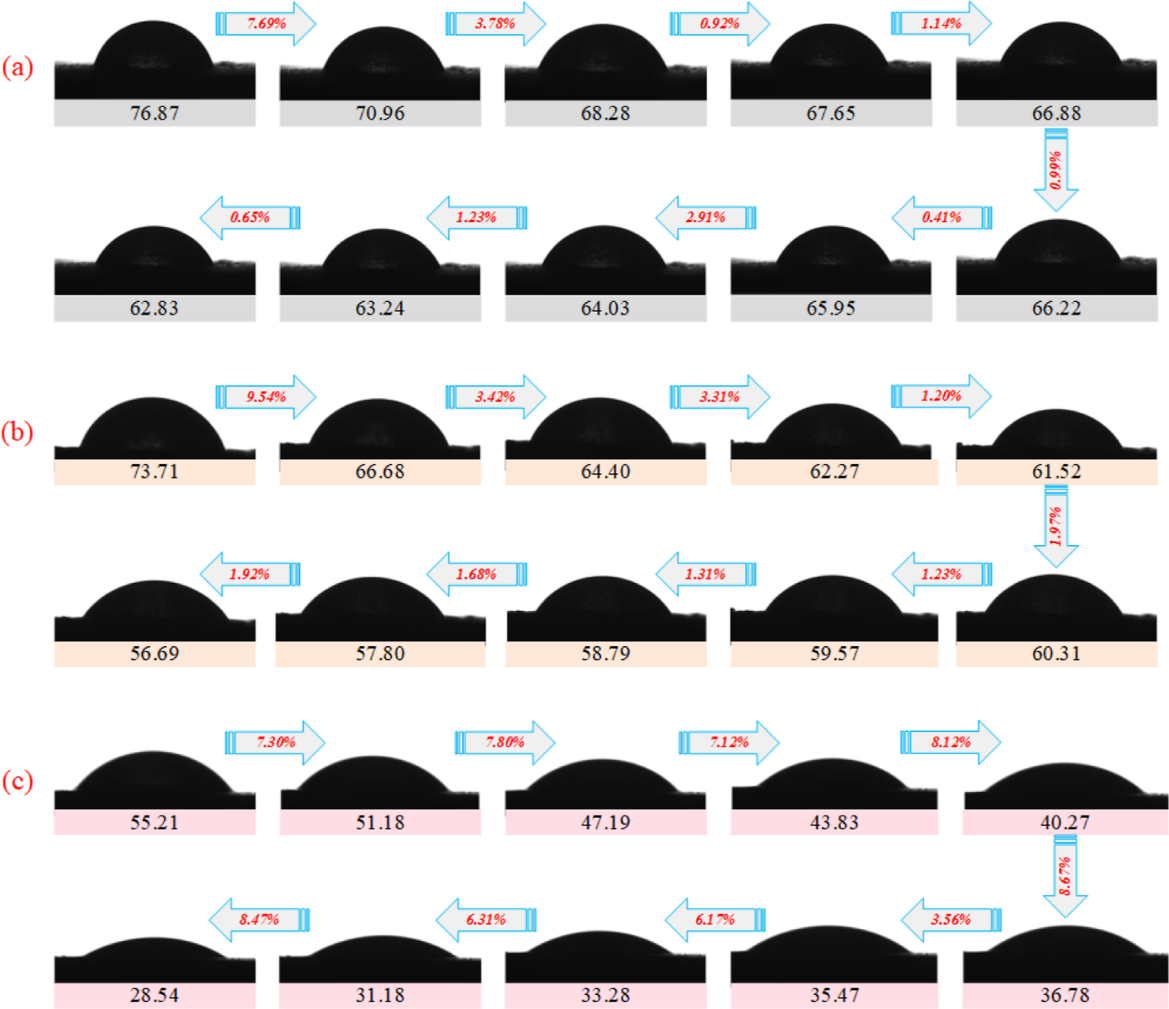
Dynamic changes of contact angles [(a) pure water, (b) pectin solution, and (c) PC-*g*-AM/TO-10 solution].

Contact angle experiments were used to consider the wetting effect of PC on coal samples before and after modification, and the interval between the measured values of contact angle was 0.02 s. The bubble diagram of the contact angle is shown in Figure 10, where the darker red color represents a larger contact angle value and the darker blue color represents a smaller contact angle value. The changes in contact angles are shown in Figure 11. From the analysis of experimental results, it can be seen that at 0.02 s, when the droplets just contact the coal flakes, the contact angle of pure water droplets on the coal flakes is the largest, with a value of 76.87°, the contact angle of PC droplets on the coal flakes is 73.71°, and the contact angle of modified PC droplets on the coal flakes is the smallest, with a value of 55.21°. It can be seen that the modified PC dust suppression droplets have the best wetting effect on coal flakes, which is reduced by 21.66° and 18.50° compared with pure water droplets and PC droplets. With the increases of contact times, the decrease rates of contact angles between pure water and PC droplets and coal flakes increase and then tend to be stable. The decrease rates of contact angles between the modified PC dust suppression droplets and coal flakes first maintain a stable trend, then begin to decrease and tend to be flat after reaching the stage of 0.12 s. At 0.20 s, when the droplets have continuously wetted bituminous coal for a period of time, the contact angle of pure water droplets on coal flakes is the largest, with a value of 62.83°, the contact angle of PC dust suppression droplets on coal flakes is 56.69°, and the contact angle of modified PC droplets on coal flakes is the smallest, with a value of 28.54°. Compared with pure water droplets and PC droplets, it decreases by 34.29° and 28.15°. It can be seen that the modified PC dust suppression solution can quickly wet coal dust and keep the coal dust in a highly wetted state.

## 4 Reaction and Dust Suppression Mechanism

As a natural additive and environmental material, PC is used as the main substance in the modification process, and the dust suppressant generated after modification is eco-friendly. PC is easy to extract and abundant, and if it can be utilized in the preparation of dust suppressants, it can bring economic effects.^1^ The structure and properties of PC can be optimized by modification.

**Figure 12:**
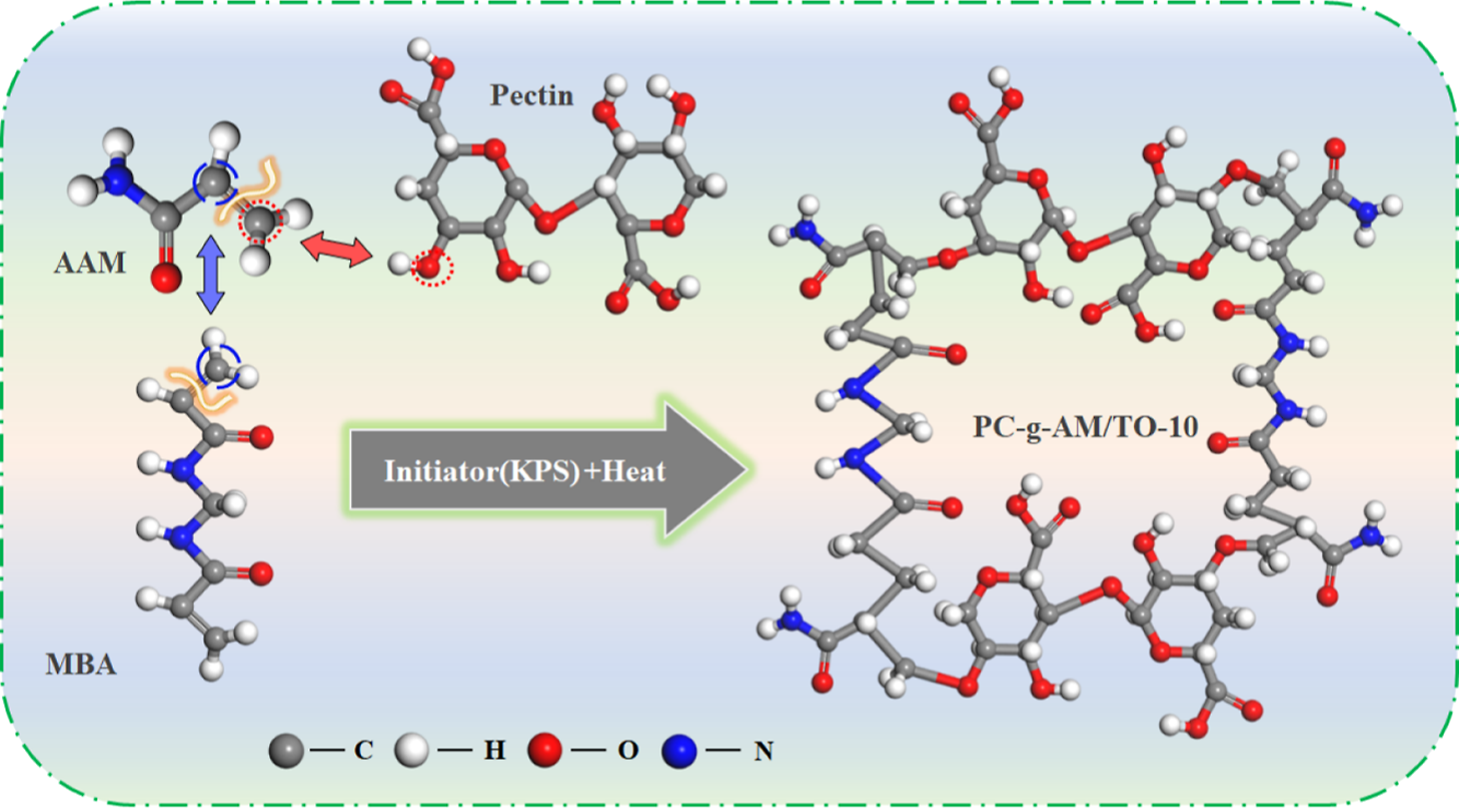
Graft copolymerization mechanism diagram.

The hydroxyl groups in PC break under the action of a high temperature and initiator, as shown in Figure 12. The double bonds in AM and MBA molecules break after being introduced into the structure, and the AM connects with the active graft sites on the PC macromolecule, which plays a key role in building the grafting backbone.^57^ The MBA acts as a bridge connecting macromolecules, which can effectively promote the growth of the grafted main chain.^30^ Surfactants are added to the product to improve the wettability of the modified dust suppressant.

**Figure 13:**
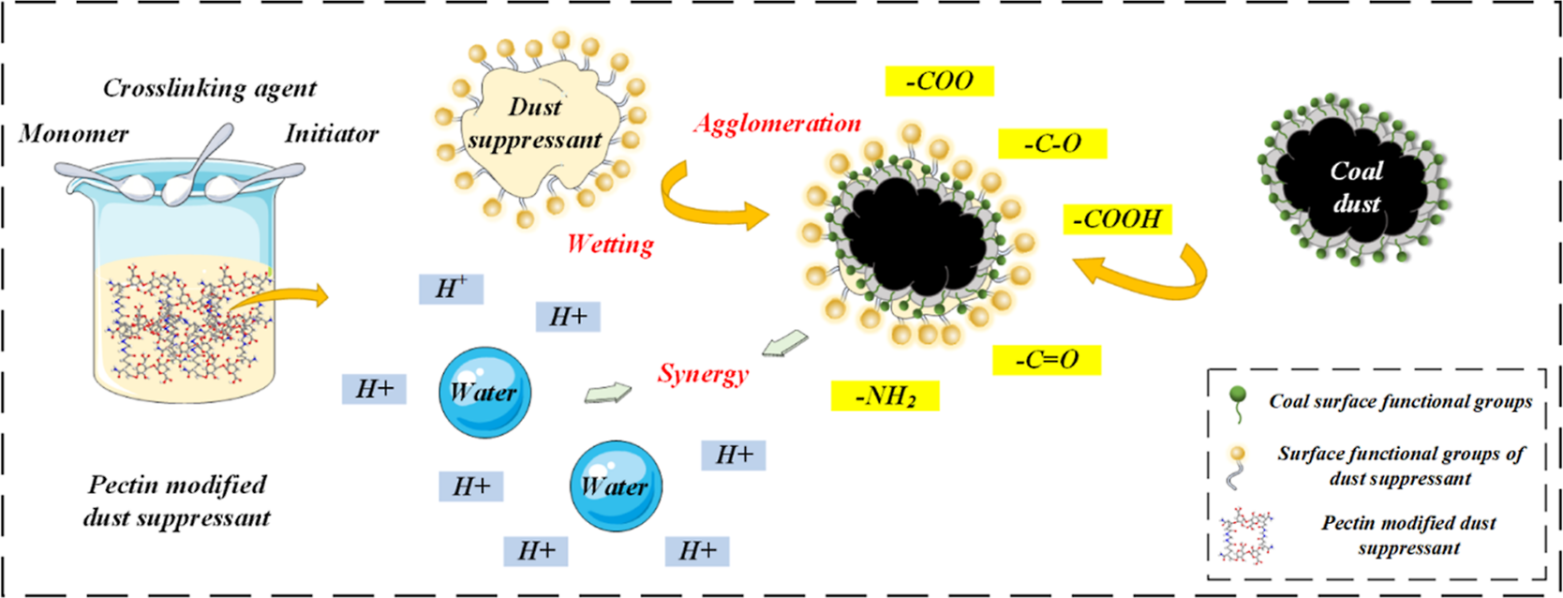
Dust suppression mechanism of solutions after pectin modification.

Figure 1a,b shows the dust suppression effects of coal piles immersed in water and dust suppressant. The coal surface after water treatment shows that a large amount of coal dust adheres to the surface, which is loose, easy to break, and can diffuse with the wind. After being soaked in dust suppressant, the surface changes greatly, and the overall structure shows a smooth and dense colloidal shape.^28^ The whole coal pile can be picked up by a tweezer, which proves that the coal dust is wetted and wrapped by the dust suppressant.^14,46^ The coal dust is tightly bound to each other and does not spread easily, which proves that the dust suppressant can play a significant role in wetting and coalescing coal dust.^26^ The dust suppression mechanism of the modified product from the molecular level is shown in Figure 13. The hydrophobic sites on the surface of coal dust are replaced by different hydrophilic groups after the dust suppressant acts with coal, which leads to the effective improvement of the coal wettability.^58,60,61^ In addition, due to the formation of free radicals in the graft copolymerization process, the presence of free H^+^ in the dust suppressant solution can promote the coal dust to attract water molecules.^51^ With the introduction of PC-modified dust suppressant into the coal, the hydrophobic end of the dust suppressant is adsorbed on the hydrophobic sites of the coal surface, which increases the hydrophilic sites on the coal surface and can attract more water molecules.^12,49^ After treatment with the dust suppressant (PC-*g*-AM/TO-10), the wettability of coal is improved.

## 5 Conclusions

In this work, dust suppressant PC-*g*-AM/TO-10 was prepared by modifying the PC. The overall conclusions are as follows:

(1) The optimal value range of each factor in the modification scheme was determined by single factor experiment, and the optimal modification scheme was finally determined by combining BBD experimental scheme and RSM optimization analysis: AM, KPS, MBA, and reaction temperature were 3.20 g, 0.20 g, 0.15 g and 92 °C, respectively.
(2) FTIR spectra confirmed that during the whole modification process, C=C and hydroxyl groups were destroyed under the action of KPS, and NH_2_ and C–O groups were introduced into the final dust suppression product. The XPS spectrum showed that the C=O and COO/COOH groups in modified PC increased by 17.69% and 10.58% compared with the original PC. The modification reaction can introduce hydrophilic groups into the structure, thus improving the wettability of PC significantly.
(3) Through SEM images, it is confirmed that the modified PC is effective in wetting and condensing coal dust. The results of contact angle experiments showed that the modified PC droplets had the best wetting effect on bituminous coal, and at 0.02 s, when the droplets just contacted the coal flakes, the wetting effect was reduced by 21.66° and 18.50° compared with pure water droplets and PC droplets, respectively. At 0.20 s, when the droplets continuously wetted the bituminous coal for a period of time, they decreased by 34.29° and 28.15° compared with pure water droplets and PC droplets, respectively.

## Nomenclature

PC: Pectin
RSM: Response surface methodology
AM: Acrylamide
KPS: Potassium persulfate
MBA: *N*,*N*′-methylene bis(acrylamide)
BBD: Box–Behnken design
FTIR: Fourier transform infrared spectroscopy
XPS: X-ray photoelectron spectroscopy
SEM: Scanning electron microscope
ANOVA: Analysis of variance
